# Transfer and Metabolism of Cortisol by the Isolated Perfused Human Placenta

**DOI:** 10.1210/jc.2017-02140

**Published:** 2017-11-16

**Authors:** Laura I. Stirrat, Bram G. Sengers, Jane E. Norman, Natalie Z. M. Homer, Ruth Andrew, Rohan M. Lewis, Rebecca M. Reynolds

**Affiliations:** 1 Tommy’s Centre for Maternal and Fetal Health, MRC Centre for Reproductive Health, University of Edinburgh, Edinburgh EH16 4TJ, United Kingdom; 2 Bioengineering Science Research Group, Faculty of Engineering and the Environment, University of Southampton, Southampton SO17 1BJ, United Kingdom; 3 Institute for Life Sciences, University of Southampton, Southampton SO17 1BJ, United Kingdom; 4 Mass Spectrometry Core, Edinburgh Clinical Research Facility, University of Edinburgh EH16 4TJ, Edinburgh, United Kingdom; 5 University/BHF Centre for Cardiovascular Science, University of Edinburgh, Edinburgh EH16 4TJ, United Kingdom; 6 Faculty of Medicine, University of Southampton, Southampton S016 6BD, United Kingdom

## Abstract

**Context:**

Fetal overexposure to glucocorticoids *in utero* is associated with fetal growth restriction and is postulated to be a key mechanism linking suboptimal fetal growth with cardiovascular disease in later life.

**Objective:**

To develop a model to predict maternal-fetal glucocorticoid transfer. We hypothesized placental 11-*β*-hydroxysteroid dehydrogenase-type 2 (11*β*-HSD2) would be the major rate-limiting step in maternal cortisol transfer to the fetus.

**Design:**

We used a deuterated cortisol tracer in the *ex vivo* placental perfusion model, in combination with computational modeling, to investigate the role of interconversion of cortisol and its inactive metabolite cortisone on transfer of cortisol from mother to fetus.

**Participants:**

Term placentas were collected from five women with uncomplicated pregnancies, at elective caesarean delivery.

**Intervention:**

Maternal artery of the isolated perfused placenta was perfused with D4-cortisol.

**Main Outcome Measures:**

D4-cortisol, D3-cortisone, and D3-cortisol were measured in maternal and fetal venous outflows.

**Results:**

D4-cortisol, D3-cortisone, and D3-cortisol were detected and increased in maternal and fetal veins as the concentration of D4-cortisol perfusion increased. D3-cortisone synthesis was inhibited when 11-*β*-hydroxysteroid dehydrogenase (11*β*-HSD) activity was inhibited. At the highest inlet concentration, only 3.0% of the maternal cortisol was transferred to the fetal circulation, whereas 26.5% was metabolized and 70.5% exited via the maternal vein. Inhibiting 11*β*-HSD activity increased the transfer to the fetus to 7.3% of the maternal input, whereas 92.7% exited via the maternal vein.

**Conclusions:**

Our findings challenge the concept that maternal cortisol diffuses freely across the placenta and confirm that 11*β*-HSD2 acts as a major “barrier” to cortisol transfer to the fetus.

## Teaser

Placental cortisol metabolism and transfer was studied using tracers and computational modeling. This indicated that the placenta presents both metabolic and physical barriers to cortisol transfer.

Cortisol, the principal circulating glucocorticoid hormone in humans, is essential for normal fetal development and tissue maturation. Fetal overexposure to glucocorticoids *in utero* is associated with intrauterine growth restriction (1) and is postulated to be a key mechanism linking suboptimal fetal growth with increased risk of cardiovascular disease in later life (2). Better knowledge of the factors regulating cortisol transfer to the fetus is essential to understand the pathophysiology of fetal growth restriction and is also relevant for prescribing of antenatal steroids, which are widely used in clinical management of women at threat of preterm birth.

Maternal circulating cortisol levels rise threefold during pregnancy (3). Although glucocorticoids are lipophilic and thus are believed to freely cross the placenta, fetal cortisol levels are 5- to 10-fold lower than maternal levels (4) due to the activity of the placental enzyme 11-*β*-hydroxysteroid dehydrogenase-type 2 (11*β*-HSD2) (5–7), which catalyzes the conversion of active cortisol into inactive cortisone. In human placenta, 11*β*-HSD2 is localized to the syncytiotrophoblast (7), which is the primary barrier between the mother and the fetus and thus prevents glucocorticoids accessing placental cells and the fetal compartment (8). Indeed, placental 11*β*-HSD2 has been suggested to inactivate the majority of maternal glucocorticoids passing to the fetus in rodents (9) and in humans (10). 11-*β*-hydroxysteroid dehydrogenase-type 1 (11*β*-HSD1), which regenerates cortisol from inactive cortisone, is undetectable in the syncytiotrophoblast, but is localized in the extravillous trophoblasts (situated near maternal circulation) and endothelial cells lining fetal capillaries in terminal villi (11). Whether the activity of placental 11*β*-HSD1 regenerates a substantial amount of cortisol or contributes significantly to maternal or fetal circulations is not well understood. With a number of studies demonstrating links between placental glucocorticoid transfer, sensitivity, and metabolism and adverse outcomes in infancy, childhood, and adolescence (12, 13), understanding of the regulatory mechanisms and rate-limiting steps of maternal-fetal cortisol transfer is essential to identify whether there are any options for targeted intervention to improve pregnancy outcomes.

Studies using the *ex vivo*, dual-perfused, placental perfusion model together with computational modeling have generated new mechanistic insights into placental amino acid and lipid transfer from mother to fetus (14–16). In the current study, we used this combined experimental and computational modeling approach to develop a model to explore placental cortisol metabolism and transfer and its regulation. We hypothesized that activity of placental 11*β*-HSD2 would be the major rate-limiting step in maternal cortisol transfer to the fetus.

## Methods

Five term placentas from women with uncomplicated pregnancies were collected on ice immediately after delivery by elective caesarean section at the Royal Infirmary of Edinburgh with ethical approval (REC09/S0704/3) and written informed consent. Elective caesarean sections were performed between 39 to 40 weeks of gestation.

### Placental perfusions

Placentas were perfused using the methodology of Schneider *et al*. (17) as adapted in a previous study (18). Nonrecirculating maternal and fetal circulations were established in an isolated cotyledon within 30 minutes of delivery. The fetal circulation and maternal intervillous space were perfused with a modified Earle’s bicarbonate buffer (EBB: 5 mmol L^–1^ glucose, 1.8 mmol L^–1^ CaCl_2_, 0.4 mmol L^–1^ MgSO_4_, 116.4 mmol L^–1^ NaCl, 5.4 mmol L^–1^ KCl, 26.2 mmol L^–1^, NaHCO_3_, 0.9 mmol L^–1^ NaH_2_PO_4_), with Heparin (25,000 units/L; Fannin, Northamptonshire, United Kingdom) and bovine serum albumin (Fraction V, 98%, 2 g/L; Sigma, Dorset, United Kingdom) added. Maternal perfusate was equilibrated with 95% air and 5% CO_2_ and fetal perfusate with 95% N_2_ and 5% CO_2_ (British Oxygen Company, Manchester, United Kingdom). Maternal circulation was at 14 mL/min and fetal circulation at 6 mL/min using a peristaltic pump (Watson-Marlow, Falmouth, United Kingdom).

Approximately 2 mL of venous perfusate was collected from the maternal and fetal venous outflows, at 5-minute intervals. Fetal artery pressure was maintained between 40 and 70 mm Hg, and fetal venous return was >95%. At the end of the experiments, the perfused mass was identified on the “maternal side” by slight blanching. The perfused placental cotyledon was weighed. Cotyledon volume was calculated on the basis of 1 mL per g tissue. Samples of maternal and fetal perfusate fluid, unperfused tissue, and perfused tissue were stored at –80°C until analysis.

### Use of deuterated tracers to investigate cortisol metabolism

**Figure 1.**
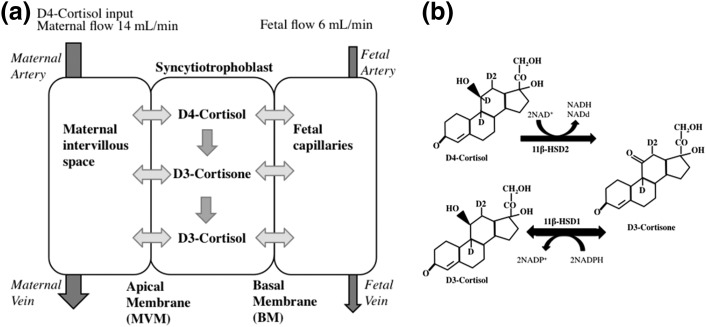
Model schematic and metabolism of deuterium-labeled glucocorticoids. (a) Model schematic showing the three compartments (maternal, syncytiotrophoblast, and fetal) distinguished in the model. It is assumed that transfer between compartments is by simple diffusion, whereas metabolic conversion between cortisol and cortisone takes place in the syncytiotrophoblast (equations 1 to 6, see “Methods” section). The input concentration of D4-cortisol in the maternal compartment varies over time according to the experimental protocol, whereas the input concentration in the fetal compartment is zero at all times. The output concentrations of the maternal and fetal compartments from the model can be compared with the experimental data. D4-Cortisol is inactivated by 11*β*-HSD2 to D3-cortisone, with the loss of the deuterium on C11. (b) 11*β*-HSD1 regenerates D3-cortisol from D3-cortisone, with the addition of an unlabeled hydrogen.

Cortisol metabolism by 11*β*-HSD enzymes and transport between the maternal and fetal circulations was investigated using the stable isotope deuterium (D)-labeled tracer, [9,11,12,12 ^2^H_4_]-cortisol “D4-cortisol” (19), which is converted to [9,12,12 ^2^H_3_]-cortisone “D3-cortisone” by 11*β*-HSD2. Measurement of [9,12,12 ^2^H_3_]-cortisol “D3-cortisol,” which is regenerated from D3-cortisone can be used to assess activity of 11*β*-HSD1 (Fig. 1). After an initial “washout” period of 30 minutes, D4-cortisol (Steraloids, Newport, RI) was perfused into the maternal circulation with stepped increases in concentrations of 20, 200, and 800 nM every 30 minutes. The 800 nM D4-cortisol concentration was considered to be representative of circulating maternal cortisol levels in the third trimester (20). The hydroxysteroid dehydrogenase inhibitor carbenoxolone (Sigma, United Kingdom) was added to the perfusion solution in addition to 800 nM D4-cortisol in the final 30 minutes at a concentration of 1000 nM, as informed by a previous study (10).

### LC-MS/MS quantification

Endogenous (cortisol, cortisone) and deuterated (D4-cortisol, D3-cortisone and D3-cortisol) glucocorticoids were measured simultaneously by liquid chromatography tandem mass spectrometry (LC-MS/MS) using a Waters Acquity UPLC (Manchester, United Kingdom) liquid chromatography system followed by mass spectral detection on an ABSciex QTRAP 5500 (Warrington, United Kingdom) operated in positive electrospray ionization mode. Mass spectral conditions are described in Supplemental Table 1 in conjunction with ion spray voltage (5500 V) and source temperature (700°C).

### Perfusate fluid extraction

Following enrichment of perfusate (500 µL) with the internal standard epi-cortisol (10 ng; Steraloids) and dilution with water (500 µL), analytes were extracted using a Sep-Pak C18 40 mg 96-well plate (Waters, Manchester, United Kingdom). Plates were primed with methanol (1 mL) and then EBB (1 mL), samples (500 µL) were loaded, and plates washed with water (1 mL). Analytes were eluted from the plate using acetonitrile (1 mL) directly into a 2-mL-deep well collection plate (Waters, United Kingdom). Eluants were dried under oxygen-free nitrogen (60°C) using a 96-well dry-down apparatus and reconstituted in mobile phase (30:70, methanol:water; 100 µL).

### Tissue extraction

Placental tissue (200 mg) was homogenized in 3 mL methanol:water (7:2) and enriched with internal standard epi-cortisol (10 ng, as previously stated) before being centrifuged at 3200*g* for 45 minutes at 4°C. Supernatant was transferred to a clean glass vial and dried under oxygen-free nitrogen (60°C) and reconstituted in water (5 mL). Analytes were extracted using Sep-Pak C18 360 mg Classic Cartridges (Waters). Cartridges were primed with 100% methanol (5 mL) followed by water (5 mL). Samples were added to cartridges and allowed to flow through with gravity. Cartridges were washed with water (5 mL), and analytes were eluted with 100% methanol (2 mL) into a 3.5-mL glass vial. Eluants were dried down under oxygen-free nitrogen (60°C) and reconstituted in 100 µL mobile phase.

### LC-MS/MS

Samples in the auto-sampler were maintained at 10°C. Analytes were separated at 40°C on an ACE Excel C18-AR column (100 × 2.1 mm, 1.7 um; Hichrom Limited, Berkshire, United Kingdom) at a flow rate of 0.5 mL/min. Samples in the auto-sampler and sample manager were maintained at 10°C. Starting with 70% water with 0.1% formic acid (solution A) and 30% acetonitrile with 0.1% formic acid (solution B), this was maintained for 4 minutes followed by a 1-minute linear rise to 60% solution B, and a subsequent rise to 90% solution B, before restoring to 30% solution B at 6.1 minutes. This condition was sustained for 1 minute to re-equilibrate.

The interassay precision of D4-cortisol in perfusate fluid was 3.6% to 11.6%, and interassay accuracy was 93% to 103%. Interassay precision of D3-cortisol in perfusate fluid was 8.8% to 17.3%, and interassay accuracy was 98% to 101%. For placental tissue samples (which were all analyzed on the same day), intra-assay precision was 7.0% for D4-cortisol and 6.4% for D3-cortisol.

The peak areas of deuterated steroids were corrected for the abundances of naturally occurring isotopomers at baseline. In addition, the peak area of D4-cortisol was corrected for interference from the M+4 isotopologue of cortisol and the M+1 isotopologue of D3-cortisol. There was no available standard for D3-cortisone, so concentrations were estimated using the calibration curve for cortisone and the “fold-change” or “units / mL” rather than concentration calculated. The peak area of D3-cortisol was corrected for interference from the M+3 isotopologue of cortisol.

### Data analysis

Deuterated hormone levels were adjusted for flow rate and were normalized to tissue weight of the perfused cotyledon. D4-cortisol and D3-cortisol were reported in ng, and in the absence of a standard for accurate quantification, D3-cortisone was measured in arbitrary units.

### Computational model for placental transfer

A compartmental modeling framework was adopted to model the placental transfer of cortisol and cortisone in the *ex vivo* perfusion experiments, based on our previous work (14, 15, 21). The model distinguishes three separate physiological compartments associated with the maternal, syncytiotrophoblast, and fetal capillary volumes (Fig. 1a). Each compartment is described as well mixed. Transfer between compartments is determined by the fluxes across the apical and basal membranes (BMs) and assumed to occur by simple diffusion for both cortisol and cortisone. Metabolic conversion from cortisol to cortisone within the syncytiotrophoblast is described as unidirectional using Michaelis-Menten kinetics. Model equations were implemented in Matlab (R2016a) as outlined previously (14, 15, 21). Details of the equations that resulted and model parameters are described in Supplemental Methods.

A sensitivity analysis was carried out in which the model parameters were varied with respect to the values for the reference fit. The reported changes in placental transfer predicted by the model were based on the steady-state results at the highest maternal input concentration.

## Results

### Characterization of subjects

The mean (standard deviation) maternal age was 36.4 ± 6.3 years, mean gestational length was 277 ± 2 days (39 + 4 weeks ± 2 days), and mean birth weight was 3721 ± 223 g.

### D4-cortisol, D3-cortisone, and D3-cortisol levels

**Figure 2.**
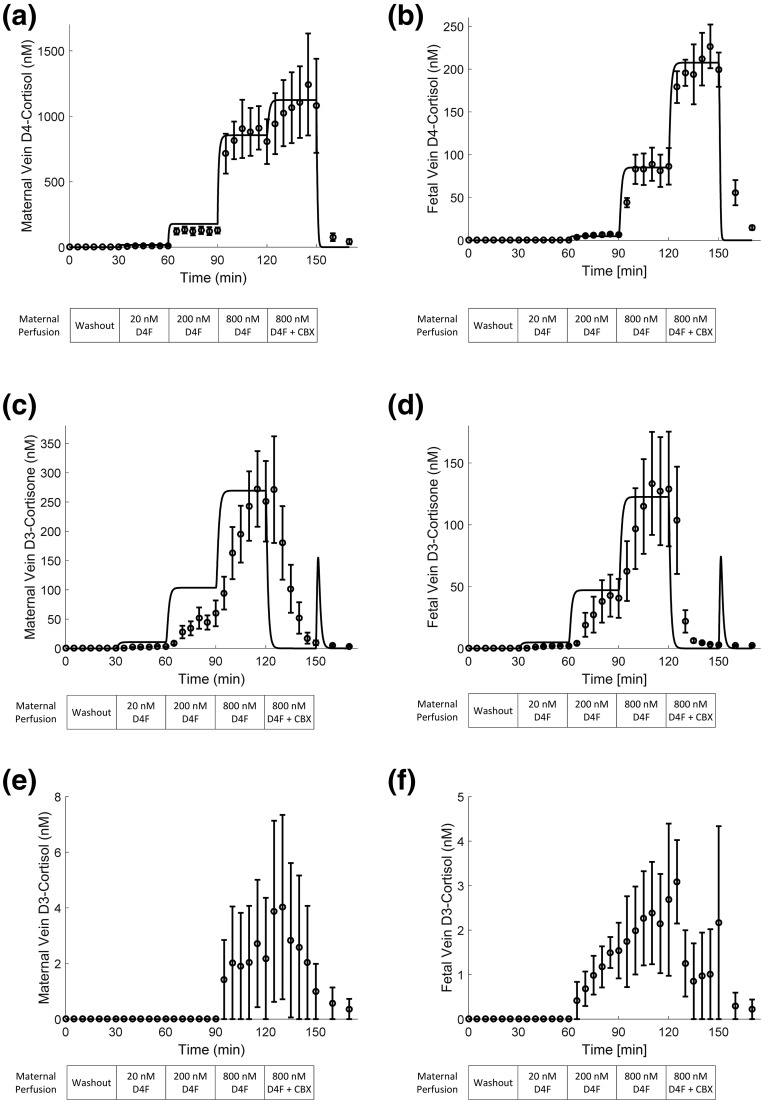
Model fit of experimental data. The maternal circulation was perfused from 0 to 30 minutes with EBB alone, maternal circulation was 0 to 30 minutes EBB alone, 30 to 60 minutes EBB + 20 nM D4-cortisol, 60 to 90 minutes EBB + 200 nM D4-cortisol, 90 to 120 minutes EBB + 80 0nM D4-cortisol, 120 to 150 minutes EBB + 800 nM D4-cortisol + 0.001 M carbenoxolone, and 150 to 170 minutes EBB alone. The appearance of D4-cortisol in the fetal circulation is consistent with free transplacental passage of D4-cortisol. Inactivation of D4-cortisol by 11*β*-HSD2 is indicated by the appearance of D3-cortisone in the maternal or fetal circulations, and cortisol regeneration from D3-cortisone is indicated by the appearance of D3-cortisol. Model fit of the experimental data for D4-cortisol in the (a) maternal and (b) fetal compartments, with a single set of parameters. Results show an excellent correspondence between model (straight line) and experiments (plotted data and error bars) (*R*^2^ = 0.99). (c, d) Model prediction of D3-cortisone in comparison with the scaled experimental data. Note the experimental units for D3-cortisone could not be directly related to concentration and have been scaled here to allow comparison of the relative changes predicted by the model. The same conversion factor was applied to both maternal and fetal D3-cortisone based on the average ratio between experimental units and computed concentrations at the highest input level (time points *t* = 110, 115, and 120 minutes). (e, f) Experimental data for D3-cortisol. Values were comparatively low and were not modeled as they do not contribute significantly to the overall mass balance. All experimental results are the average of five placentas, expressed as mean and standard error of the mean (n = 5). Abbreviations: CBX, carbenoxolone; D4F, D4-cortisol.

Fig. 2 shows the levels of D4-cortisol, D3-cortisone, and D3-cortisol (plotted data with error bars) in maternal and fetal veins increased as the concentration of D4-cortisol in the maternal artery perfusion increased. D4-cortisol (Fig. 2a and 2b) and D3-cortisone (Fig. 2c and 2d) were detected in maternal and fetal vein 5 minutes after commencement of D4-cortisol perfusion (20 nM) in the maternal artery. D3-cortisol (Fig. 2e and 2f) was detected at 95 minutes into the experiment in the maternal vein (perfusion phase: 800 nM D4-cortisol) and at 75 minutes in the fetal vein (perfusion phase: 200 nM D4-cortisol). Variation in the D3-cortisol levels reflects both the fact that D3-cortisol levels were near the limit of detection and technical considerations when collecting maternal side samples in the perfusion system where variation tends to be higher. The biggest increase in D4-cortisol and D3-cortisone levels occurred when maternal artery D4-cortisol perfusion increased from 200 to 800 nM. Levels of D3-cortisone in the maternal circulation were approximately fivefold higher than in the fetal circulation. When carbenoxolone was added to the maternal artery perfusion, D4-cortisol levels further increased in maternal and fetal veins, and D3-cortisone synthesis was completely inhibited. D3-cortisol levels were around 300-fold lower than D4-cortisol in both maternal and fetal circulations and were close to the assay limit of detection. Levels of D3-cortisol in the maternal circulation were approximately two- to threefold higher than levels in the fetal circulation. Proportionately more of the produced D3-cortisol was released into the fetal circulation than maternal circulation, when compared with the proportion of D3-cortisol released into maternal and fetal circulations. Samples of buffer obtained on completion of the “washout” phase of the experiment confirmed that there were no remaining endogenous or labeled glucocorticoids within the tubing used for the circuit.

### Placental model results

The results of the model fit of the average maternal and fetal D4-cortisol measurements demonstrated an excellent overall ability of the computational model to represent the experimental data (Fig. 2). From the model, the estimated effective membrane permeability constant $k_{MVM}$ = 0.011 L/min for the maternal-facing microvillous membrane (MVM) and $k_{BM}$ = 0.0015 L/min for the fetal-facing BM. Thus the permeability of the MVM was estimated to be 7.4 times higher than that of the BM. The estimated maximum rate capacity for the conversion of cortisol into cortisone $V_{4F\to 3E}^{max}$ = 5.0 nmol/min per cotyledon. At the highest inlet concentration, only 3.0% of the maternal cortisol input was transferred to the fetal circulation, whereas 26.5% was metabolized and the remaining 70.5% exited via the maternal vein. Inhibiting 11*β*-HSD activity increased the transfer to the fetus to 7.3% of the maternal input, whereas 92.7% exited via the maternal vein. Based on these results, it can also be seen that enzyme metabolism reduced transfer to the fetus by 59%. Note that if there were no placental barrier and no metabolism, then the maternal and fetal vein would have an output of respectively 70% and 30% of the maternal inlet, based on the difference in flow rates alone (*i.e.*, if concentrations within the placenta were perfectly mixed). The comparison between the predicted D3-cortisone and the scaled experimental data are shown in Fig. 2. It can be observed that the relative steady-state levels correspond well for the fetal D3-cortisone, whereas the maternal D3-cortisone shows some larger discrepancies. In addition, the model responds much more rapidly to changes in input conditions. In this respect, the sharp peak at *t* = 150 minutes predicted by the model is due to the absence of blocker in the washout buffer, which is assumed to take immediate effect in the model.

**Figure 3.**
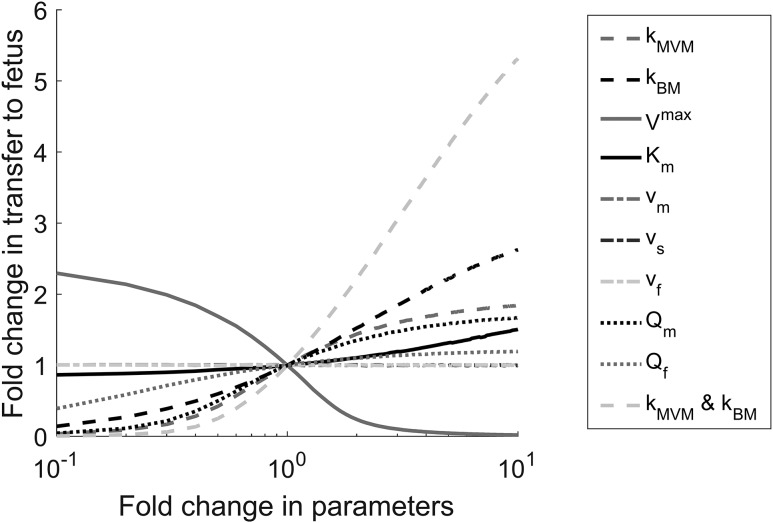
Sensitivity analysis for D4-cortisol transfer to the fetus as a function of variations in the model parameters. The model parameters were varied with respect to the values for the reference fit. The reported changes in placental transfer predicted by the model were based on the steady-state results at the highest maternal input concentration. Abbreviations: *k*_BM_, BM permeability constant; *K*_m_, Michaelis-Menton constant; *k*_MVM_, MVM permeability constant; *Q*_f_, fetal flow rate, L/min; *Q*_m_, maternal flow rate, L/min; *V*_f_, fetal compartment volume; *V*_m_, maternal compartment volume; *V*^max^, maximum rate of reaction; *V*_s_, syncytiotrophoblast compartment volume.

The results of the sensitivity analysis in Fig. 3 show that when varying single parameters, the placental transfer of cortisol was affected most by changes in $k_{BM}$, the membrane permeability of the BM, and the metabolic conversion rate of cortisol into cortisone, $V_{4F\to 3E}^{max}$. In addition, placental transfer was predicted to be moderately sensitive to $k_{MVM}$, the permeability of the MVM, and the maternal flow rate used in the experiment, $Q_{m}$. Variations in $K_{m}$ only had a small impact as the metabolism continued to operate in the saturated regimen, whereas increasing the fetal flow rate, $Q_{f}$, used in the experiment was predicted to only have a minor effect on transfer. Steady-state transfer was not sensitive to any of the compartment volumes, as expected. To evaluate the impact of the overall membrane permeability, an additional study was done in which $k_{BM}$ and $k_{MVM}$ were both varied simultaneously, demonstrating a considerably larger effect than for the permeability of each membrane separately (Fig. 3).

## Discussion

The experiments performed in this study using a deuterated cortisol tracer in the *ex vivo* placental perfusion model allowed investigation of the role of interconversion of cortisol and its inactive metabolite cortisone on transfer of cortisol from mother to fetus at term. The application of computational modeling enabled interpretation of the transfer mechanisms that underlie these processes. Our findings challenge the concept that maternal cortisol diffuses freely across the placenta, confirm that 11*β*-HSD2 acts as a major “barrier” to cortisol transfer to the fetus, and show preliminary evidence of local cortisol production within the placenta.

Addition of carbenoxolone (a potent hydroxysteroid dehydrogenase inhibitor) to the maternal artery perfusion resulted in no further production of D3-cortisone. This supports the role of 11*β*-HSD2 as a key player in the maternal barrier to fetal glucocorticoid exposure. The activity [but not messenger RNA (mRNA)] of 11*β*-HSD2 has been shown to decrease in the last 2 weeks before parturition (22). The placentas used in the experiments were obtained from elective caesarean sections at between 39 and 40 weeks gestation, so it is not known when parturition would have occurred in these pregnancies. The model allowed an estimation of the maximum capacity of 11*β*-HSD2 for conversion of cortisol to cortisone as 5.0 nmol/min per cotyledon. It is not known what the capacity of 11*β*-HSD2 would be if exposed to high levels of maternal glucocorticoids for more prolonged periods, but studies have demonstrated that 11*β*-HSD2 mRNA and activity is downregulated by maternal stress (23) and inflammatory diseases (22). Further, inhibition of 11*β*-HSD2 by maternal liquorice consumption has adverse consequences on child development (24, 25). Our study supports the premise that the adverse offspring outcomes are due to increased fetal glucocorticoid exposure, as when 11*β*-HSD2 was inhibited by carbenoxolone, transplacental passage of maternal cortisol to the fetal circulation was more than doubled.

Yet, even when 11*β*-HSD activity was inhibited using carbenoxolone, less than 10% of maternal D4-cortisol crossed the placenta in our experiments. This observation challenges the concept that cortisol freely diffuses across the placenta and suggests alternate mechanisms to protect the fetus from high maternal cortisol levels in addition to the well-described inactivation of cortisol by 11*β*-HSD2. Three adenosine triphosphate binding cassette (ABC) transporters, multidrug-resistant protein (MRP1, encoded by *ABCC1*), *p*-glycoprotein (P-gp, encoded by *ABCB1*) and breast cancer–resistant protein (BCRP, encoded by *ABCG2*), are localized to placental syncytiotrophoblast and the fetal vessel endothelium (26, 27), consistent with the potential for active transport of cortisol in and out of the placenta. Further studies are needed to investigate the contribution of ABC transporters, levels of which are known to alter across gestation (28–31), in regulating maternal cortisol transfer to the fetus and in particular to understand the kinetics of efflux transporters, which our preliminary observations suggest may also protect the fetus.

Further we observed approximately a fivefold higher D3-cortisone release to the maternal circulation compared with the fetal circulation. It also needs to be considered that the physical process of cortisol diffusion across tissues may be more challenging than has been thought previously. In particular, in the placenta, diffusion across the water-filled villous stroma may prove a barrier to cortisol diffusion. This is consistent with the observation that cortisone was preferentially released into the maternal circulation (2:1 maternal:fetal circulation) and the lower placental-to-fetal permeability calculated within the model.

A unique finding is the observation of *de novo* placental cortisol synthesis, as evidenced by the detection of D3-cortisol in both maternal and fetal circulations. Though the absolute levels of D3-cortisol were low, this regeneration of cortisol may have local paracrine roles, and increased placental 11*β*-HSD1 mRNA levels have been associated with maternal depression and with altered infant regulatory behaviors (12, 13). Further, proportionately more D3-cortisol was transferred to the fetus than D3-cortisone, which is in line with localization of 11*β*-HSD1 to the endothelium (11). The computational model provided a good overall representation of the experimental data under different experimental conditions. In general, the compartmental model showed a faster response due to the well-mixed assumption, but this did not affect the steady-state levels. The model predicted that changing membrane permeability of the BM would affect placental transfer of cortisol. Placental transfer of lipids has been reported to be increased in pre-eclampsia (32). Further studies are required to investigate whether inflammatory conditions such as pre-eclampsia and preterm labor alter the permeability of the BM and thus alter placental cortisol transfer.

Our study has several limitations. This study focused on cortisol concentrations in the maternal and fetal plasma, as these determine the gradient driving transfer. However, from a whole body perspective, it needs to be realized that the maternal plasma compartment is larger than the fetal compartment, and this would need to be taken into account for more broadly focused models. Our experiments were conducted using EBB buffer and albumin. The findings may be altered *in vivo* with the presence of corticosteroid-binding globulin, the primary binding protein for cortisol (33), and this should be considered in future studies. Including such binding effects would not affect the overall modeling results if the unbound fraction is constant in the concentration range used, but would become important if binding differs between compartments. We were also unable to accurately quantify D3-cortisone concentrations, as there are no available standards. Nevertheless, we were able to estimate fold-changes in D3-cortisone concentrations, so this should not limit interpretation of the results. A caveat of the model is that it does not account for further interconversion of D3-cortisol to D3-cortisone, although the net values of D3-cortisol quantified were very low. We did not study other pathways of cortisol metabolism such as the A-ring reductase enzymes, although Benediktsson *et al.* (10) found that the products of 5*β*-reductase or 20*α*/*β*-hydroxysteroid dehydrogenase did not coelute with cortisol or cortisone in placental perfusion studies, suggesting that these pathways may not metabolize cortisol or cortisone in the placenta. The contribution of other potential metabolism pathways, such as via carbonyl reductase 1 (34), which is located in placenta, is also unknown. Direct measurement of arterial input concentrations would also have provided additional confidence to this analysis.

Further studies using this model could investigate in more detail the contribution of the fetal circulation to maternal cortisol levels. Regeneration of cortisol from cortisone could be studied by perfusing the fetal circuit with D2-cortisone (35) and measuring the regenerated cortisol in the maternal or fetal circuits. The potential for free placental passage of cortisol from the fetal to maternal circuit could be studied by perfusing the fetal circuit with D4-cortisol and measuring D4-cortisol, D3-cortisone, and D3-cortisol in the maternal circulation. Future studies utilizing inhibitors of ABC transporters are also needed to assess their contribution to placental cortisol transport. Although technically challenging, functional studies using early and midgestation tissue would be of value, as cortisol exposure at earlier gestations is thought to influence fetal growth (36, 37). Our model may also be a helpful tool in predicting fetal effects of synthetic glucocorticoids such as dexamethasone and betamethasone, used clinically to promote fetal lung maturation when preterm delivery is anticipated.

To conclude, we have developed a model to predict maternal-fetal cortisol transfer, which can now be used in future experimental design. Further studies are now needed to refine and develop the model to improve understanding of the mechanisms underlying maternal-fetal cortisol transfer and the pathways to normal fetal growth.
