# Supplementary material for: Transfer and Metabolism of Cortisol by the Isolated Perfused Human Placenta
Source: J Clin Endocrinol Metab. 2017 Nov 16;103(2):640–8. doi: 10.1210/jc.2017-02140 (PMC5800837; doi:10.1210/jc.2017-02140)
Supplement: Supplementary file 1 [file jc.2017-02140.sm1.pdf]

Transfer and Metabolism of Cortisol by the Isolated Perfused Human Placenta

*Laura I Stirrat[1], *Bram G Sengers[2,3], Jane E Norman[1], Natalie Z.M Homer[4,5], Ruth Andrew[4,5], **Rohan M Lewis[3] and **Rebecca M Reynolds[1,5]

1. Tommy's Centre for Maternal and Fetal Health, MRC Centre for Reproductive Health, University of Edinburgh
2. Bioengineering Science Research Group, Faculty of Engineering and the Environment, University of Southampton, UK
3. Institute for Life Sciences, University of Southampton, UK.
4. Mass Spectrometry Core, Edinburgh Clinical Research Facility, University of Edinburgh, UK
5. University/BHF Centre for Cardiovascular Science, University of Edinburgh, UK

*joint first author

**joint last author

Supplementary Method

Details of the model equations and model parameters are described below.

1. Model equations

$$\frac{dC_A^m}{dt} = \frac{1}{v_m}\left(Q_m\left(C_A^{in,m} - C_A^m\right) - k_{MVM}\left(C_A^m - C_A^s\right)\right) \quad [1]$$

$$\frac{dC_A^s}{dt} = \frac{1}{v_s}\left(k_{MVM}\left(C_A^m - C_A^s\right) - k_{BM}\left(C_A^s - C_A^f\right) + J_A^{metab}\right) \quad [2]$$

$$\frac{dC_A^f}{dt} = \frac{1}{v_f}\left(k_{BM}\left(C_A^s - C_A^f\right) - Q_f C_A^f\right) \quad [3]$$

where C_A^m, C_A^s and C_A^f are the concentrations (mol/L) of solute A which can be either D4-cortisol (D4F), D3-cortisone (D3E) or D3-cortisol (D3F) in the maternal "*m*", syncytiotrophoblast "*s*" and fetal "*f*" compartment respectively. Similarly, the volumes v (L) of the different compartments are indicated with subscripts using the same notation. Q_m and Q_f (L/min) are the fluid flow rates in the maternal and fetal circulation. $C_A^{in,m}$ is the maternal inlet concentration, which is zero for all solute species except D4-cortisol. Note that the fetal inlet concentration is zero for all species and therefore has not been included. k_{MVM} and k_{BM} denote the effective overall permeability constants (L/min) for the microvillous membrane (MVM) and basal membrane (BM) including surface area. These diffusive permeability constants were assumed to be the same for all solute species. The metabolic conversion rate J_A^{metab} (mol/min) depends on the solute species as follows:

$$J_{D4F}^{metab} = -\frac{V_{4F\rightarrow 3E}^{max}\, C_{D4F}^s}{K_m + C_{D4F}^s} \qquad [4]$$

$$J_{D3E}^{metab} = \frac{V_{4F\rightarrow 3E}^{max}\, C_{D4F}^s}{K_m + C_{D4F}^s} - \frac{V_{3E\rightarrow 3F}^{max}\, C_{D3E}^s}{K_m + C_{D3E}^s} \qquad [5]$$

$$J_{D3F}^{metab} = \frac{V_{3E\rightarrow 3F}^{max}\, C_{D3E}^s}{K_m + C_{D3E}^s} \qquad [6]$$

where V^{max} (mol/min) is the maximum overall metabolic conversion rate and K_m (mol/L) is the Michaelis-Menten constant, i.e. the concentration at which half the maximum rate occurs.

2. Model parameters

The total cotyledon volume was based on the average cotyledon weight from the experiments (30.8 × 10^{-3} kg, n = 5), which was directly equated to the volume in L. The volume fractions of the maternal, syncytiotrophoblast and fetal compartments distinguished in the model were set to 34%, 15% and 7.4% respectively, as in our previous work. [14,22] The flow rates in the maternal and fetal circulations Q_m= 14 × 10^{-3} L/min and Q_f= 6 × 10^{-3} L/min were directly based on the experimental settings. To account for any discrepancies between nominal and actual values, the D4-cortisol input concentrations C_A^{in} used in the model were calculated based on the combined maternal and fetal steady state output during the blocking phase. The Michaelis-Menten constant K_m was set to 44 × 10^{-9} mol/L, based on the value for the enzyme 11β-HSD2 for cortisol. [23] In first instance the same value was adopted for both metabolic conversion steps in Equations 4-6.

2. Parameter estimation

The remaining parameters in the model were determined by fitting the experimental data. The following error criterion was defined for a certain species A and compartment j in general:

$$R_A^j = \frac{1}{\left(\bar{C}_A^{exp,j}\right)^2} \sum_{i \in T} \left(C_{A,i}^j - C_{A,i}^{exp,j}\right)^2 \qquad [7]$$

where $C_{A,i}^j$ and $C_{A,i}^{exp,j}$ are the computed and experimental concentrations at time point i, respectively, while $\bar{C}_A^{exp,j}$ is the mean of the experimental time points considered. The model was fitted to the steady state values after each change in maternal input concentration, including the blocking phase, therefore the set of time points T consisted of the last 4 time points for each different input phase (16 time points in total).

The D3-cortisol concentrations measured experimentally were 300 times smaller compared to D4-cortisol and did not contribute significantly to the overall mass balance. Therefore the conversion to D3-cortisol was neglected in the parameter estimation by setting $V_{3E \to 3F}^{max}$ to zero. In addition, the measured D3-cortisone values could not be directly related to concentration. Therefore D3-cortisone was not fitted, but instead the experimental values for D3-cortisone were scaled to allow comparison of the relative changes predicted by the model. Thus, only the D4-cortisol values in the maternal and fetal compartments (averaged over 5 placentas) were fitted according to the following overall error criterion:

$$R_{tot} = R_{D4F}^{m} + R_{D4F}^{f} \qquad [8]$$

In total 3 parameters were fitted, the membrane permeability constants k_{MVM} and k_{BM} and the maximum rate of conversion from cortisol to cortisone $V_{4F \to 3E}^{max}$. Time integration of Equations 1-3 was performed in Matlab (R2016a) using the *ode45* function (Runge-Kutta (4, 5) method). Parameter estimation by minimising Eq. 8 was implemented using the *fminsearch* function (Nelder-Mead method). Initial parameter estimates were varied to verify that the algorithm converged to a unique solution.

Supplementary Tables

Supplementary Table 1 Mass spectral conditions for analysis of analytes and internal standards by positive ion electrospray ionisation

	Molecular Weight (amu)	Precursor ion (*m*/*z*)	Product ion (*m/z*) Quan; Qual	Declustering Potential (V)	Collision energy (V) Quan; Qual	Cell exit potential (V) Quan; Qual
**ANALYTES**						
D4-cortisol	367.0	367.0	121; only one	121	25	20
D3-cortisol	366.0	366.0	121.1; only one	121	25	20
D3-cortisone	363.2	364.2	164.0; only one	166	31	14
**INTERNAL STANDARDS**						
Epi-cortisol	363.2	363.2	121.0; 77.0	131	29; 101	14; 14

Abbreviations : Atomic mass units (amu) Quan (quantifier ion), Qual (qualifier ion), V (volts)

Supplementary Table 2 Inter-assay precision and accuracy

Concentrations of cortisol, cortisone, D4-Cortisol and D3-Cortisol were determined using calibration curves. Fourteen standards were prepared in 500 µL EBB (range of concentrations 0.1 ng – 400 ng) enriched with internal standards (10 ng) along with blank samples were diluted in 500 µL of water and processed using the same extraction method and analysis conditions as perfusate samples. Standard curves were plotted by calculating the peak area (analyte peak area / internal standard peak area). Weighting of 1/x and was applied to form standard curves of best fit with a regression coefficient above 0.99. The ion ratio (quantitative ion/qualitative ion) of the analytes was calculated using MultiQuant software and results were not considered acceptable if the ratio was greater than 20% of the ratio of the standards. Inter-assay fourteen point standard curve validation (n=6 different day respectively) was used to assess the limits of quantification of accuracy and precision for each analyte. Precision was based on the percentage relative standard deviation (%RSD), which was calculated using peak area ratios. Tissue sample* is intra-assay (amount, ng for tissue replicates (n=6). Inter-assay was not performed for tissue samples, as all tissue samples were analysed on the same day. Low values are the limit of quantification for each analyte.

		**Concentration (ng/200 µL perfusate or mg tissue*): mean (SD)**	**Precision (% RSD)**	**Accuracy (%)**
**D4-Cortisol**	Low (0.2)	0.21 (0.02)	10.5	103
	Mid (50)	46.7 (1.7)	3.6	93
	High (400)	396.6 (43.4)	11.6	93
	Tissue Sample*	8.3 (0.6)	7.0	
**D3-Cortisol**	Low (0.1)	0.1 (0.02)	17.3	98
	Mid (10)	10.4 (0.9)	8.8	104
	High (20)	20.2 (2.0)	10.1	101
	Tissue Sample*	0.6 (0.04)	6.4	

Abbreviations: EBB (Earle’s Bicarbonate Buffer), SD (Standard Deviation), RSD (Relative Standard Deviation)
